# Construction Notes

**Published:** 1897-06-19

**Authors:** 


### CONSTRUCTION NOTES.

### The Park Hospital, Hither Green.

This hospital is the largest of three infectious diseases hospitals erected, or in course of erection, by the Metropolitan Asylums Board, containing, as it does, beds for 548 patients. There are eight two-storied pavilions for scarlet fever, four for diphtheria, and six isolation pavilions for other cases. The central administration buildings consist of the kitchen block, steward's stores, and residences for the servants; while on either side are the steward's house and that of the assistant medical officer. From these radiate all the various buildings for patients, as well as the nurses' home, which is on the highest point to the south-west. Open but covered corridors connect all portions of the hospital, and beneath these are subways for all pipes, wires, &c. Other detached buildings comprise the medical superintendent's house, the lodge, the offices, and the waiting and discharge rooms, mortuary, &c. The buildings cover a site of nearly twenty acres, and some idea of their extent may be gathered from the fact that there are upwards of five miles of drainage within the curtilage. The works are making rapid progress, nearly all scaffolding being removed, while many buildings are approaching completion. The general contractors are Messrs. Leslie and Co., of Kensington Square. The architect is Mr. Edwin T. Hall, F.R.I.B.A., of 57, Moorgate Street, London.

### The New Pathological Institute at Glasgow.

The Western Infirmary and the medical school of Glasgow University may well be proud of the Pathological Institution which has been recently opened in their city. The study of pathology is of such infinite importance as a handmaid of medicine and surgery that every encouragement and facility should be offered to those who work in this field, and in the new buildings Professor Coats has, to judge from the plans and descriptions we have seen, as well-equipped a workshop as anyone could desire. The building consists of two floors, and is lighted throughout by electricity. On the ground floor are a chemical laboratory, a balance room, rooms for the professor and his assistants, and a laboratory for private research. There is a museum, equipped from the existing university and infirmary pathological departments, a post-mortem theatre, and a mortuary; neither is reverence for the dead neglected, for there is a mortuary chapel. Besides these, there are a lecture theatre, with a photographic dark room attached, various workshops, and the engine-room. On the upper floors are the upper portions of the two theatres and the museum, two practical class-rooms, and a bacteriological laboratory. The building cost some £15,000, and was designed by Mr. John Burnet.

**New Accident Hospital, Alloa, N.B.**

This new building is to be erected on a site immediately to the west of the existing hospital, from plans prepared by Mr. R. A. Bryden, architect, Glasgow. There will be two entrance gates on a new road leading to the hospital buildings. The main building will consist of a central administrative block and two wings. Each of the wings will contain one ward for six beds, and one ward for three beds. also two private wards for one bed each. All these wards will face the south. The wards will be approached from the central part of the building by wide corridors. Ward sculleries are also provided, entering off the corridors. Observation windows are arranged off the corridors. The floors of the wards are of pitch pine, and all corners are rounded. The lower walk of the corridor are lined with tiles, and each corridor has an exit porch for convalescent patients, obtaining access to the grounds in rear of the buildings. The central part of the building contains, toward the front, the entrance vestibule, doctor's room, men's and women's day-room, behind which are placed the operating room, doctor's store, kitchen and scullery service, pantry, and matron's store-room, &c. The central building is ten stories in height, and on the upper floor contains matron's rooms and seven bedrooms. In the rear of the main hospital building will be erected the offices required, comprising washing-house, laundry, boiler-shed, store for patients' clothing, mortuary, and post-mortem room.

